# Editorial: Reviews in psychiatry 2022: psychopharmacology

**DOI:** 10.3389/fpsyt.2024.1382027

**Published:** 2024-02-28

**Authors:** Maris Taube

**Affiliations:** ^1^ Department of Psychiatry and Narcology, Riga Stradiņš University, Riga, Latvia; ^2^ Department for Depression and Crisis, Riga Center of Psychiatry and Narcology, Riga, Latvia

**Keywords:** antidepressants, depression, schizophrenia, sleep, vortioxetine, metformin, microdosing

Psychopharmacological treatments play a leading role in psychiatry, especially in the treatment of schizophrenia (1), bipolar affective disorder, and unipolar depression. The biopsychosocial model, in which medication and its use play an important role, is now accepted and fundamentally unquestioned in psychiatry (2). Furthermore, the process of deinstitutionalization in psychiatry, which enabled patients with severe psychiatric disorders to move from hospital- to community-based care, was primarily made possible by the development of psychopharmacology and the possibility of controlling psychotic, affective symptoms with medication (3).

Unfortunately, medicines do not always have the desired effect, and patients may stop taking them due to side effects (4) or other reasons (5). Given the essential role of medication in psychiatry, extensive scientific research into its use is warranted, not least in the search for new applications.

Four of the five authors of the articles published in ‘Research Topic Reviews in Psychiatry 2022: Psychopharmacology’ chose a systematic review as their research method. This method improves the quality of scientific conclusions by filtering out studies of low quality (6). It is particularly useful when a large number of studies are available and a narrow topic is investigated.

Three articles (Lam et al., Zhang et al., and Petranker et al.) from the Research Topic focused on treatment options for depression.

Lam et al. analyzed antidepressant prescribing behavior by examining 37 quantitative studies. The most commonly prescribed antidepressants were from serotonin reuptake inhibiter (SSRIs) groups, although serotonin-norepinephrine reuptake inhibitors (SNRIs) currently play a more significant role. Furthermore, Lam et al. in the article speculated about the prescription of tricyclic antidepressants (TCAs), which is second largest prescribed group of antidepressants, exceed recommended guidelines and be used in secondary care settings.

In a meta-analysis by Zhang et al., 20 studies on the use of vortioxetine for the treatment of depression in adults were evaluated. Vortioxetine exhibited good efficacy and tolerability compared to placebos (and equivalent efficacy to SNRIs and SSRIs) and better tolerability than SNRIs. One specific role for vortioxetine could be to improve cognitive function in depressed patients, which would provide better opportunities for targeted drug selection in a clinical setting. Vortioxetine has also shown potential for treating depression in dementia patients (7), which is in line with the study by Zhang et al.

Psychiatric research is actively pursuing the development of new approaches for the treatment of psychiatric disorders, especially depression, and the use of psychedelics is one such area. In the article by Petranker et al., the authors called for a critical assessment of the research quality in the field of psychedelics, as (in their view) the results of 15 published studies did not allow for clear conclusions to be drawn concerning the efficacy or safety of this new therapeutic approach.

Two articles in the Research Topic explored important and specific aspects of schizophrenia treatment. Carlo et al. included 70 articles that met the inclusion criteria for their systematic review and meta-analysis. Somnolence, sedation, and insomnia were the most common adverse effects of antipsychotics on sleep. Amisulpiride, clozapine, flupentixol, chlorpromazine, and perospirone showed better safety profiles, while risperidone, haloperidol, perphenazine, and ziprasidone were the least safe for sleep. The authors drew attention to the need for further studies, more detailed analyses of long-acting injectables (LAIs) antipsychotics impact on sleep, and the need for appropriate dosing.

Battini et al., in their systematic review, meta-analysis, and meta-regression, addressed serious topics in the treatment of schizophrenia patients, including weight gain - a metabolic disturbance more typically seen with the new generation of antipsychotic drugs. The authors attempted to uncover whether metformin could help reduce antipsychotic-medication-induced weight gain and improve cognitive and other functions in schizophrenia patients. The authors concluded that metformin has the potential to improve cognitive abilities and other symptoms in schizophrenia patients, as well as reduce weight gain, a common side effect of antipsychotic medications.

Virtually all authors of the systematic reviews pointed to the need to improve the quality of research methodologies such that they contribute to research without being excluded from systematic reviews.

## Author contributions

MT: Conceptualization, Writing – original draft, Writing – review & editing.
